# Evidence-based psychology

**DOI:** 10.1590/S1516-31802011000200001

**Published:** 2011-03-03

**Affiliations:** I PhD. Researcher and Professor of Postgraduate Studies, Discipline of Evidence-Based Medicine and Emergency Medicine and Brazilian Cochrane Center, Universidade Federal de São Paulo — Escola Paulista de Medicina (Unifesp-EPM), São Paulo, Brazil.; II PhD. Adjunct Professor and Coordinator of the Psychology Course, Universidade Presbiteriana Mackenzie.; III MD, MSc, PhD. Nephrologist. Director of the Brazilian Cochrane Center and Titular Professor of Evidence-Based Medicine and Emergency Medicine, Universidade Federal de São Paulo — Escola Paulista de Medicina (Unifesp-EPM), São Paulo, Brazil.

Evidence-based clinical practice has become the most important scientific strategy for providing clarifications for patients, in all specialties involved in health promotion and care. In 2005, in the light of demands to follow suit, the American Psychological Association (APA)^1^ published results from a presidential task force under the title "Evidence-Based Clinical Practice in Psychology", which emphasized the importance of scientific evidence in psychological practice, alongside clinical experience and patients' individual preferences and characteristics. The APA pointed out that there was an urgent need to bring in scientific evidence within psychology: "Evidence-based practice in psychology is therefore consistent with the past twenty years of work in evidence-based medicine, which advocated for improved patient outcomes by informing clinical practice with relevant research. The use and misuse of evidence based principles in the practice of health care has affected the dissemination of health care funds, but not always to the benefit of the patient. Therefore, psychologists, whose training is grounded in empirical methods, have an important role to play in the continuing development of evidence based practice and its focus on improving patient care".^1^ The search for scientific evidence to underpin psychotherapeutic practice also involves prevention-related objectives, given that early treatment may often avoid or diminish the psychological distress that results from prolongation or worsening of psychological disorders.

*Evidence-based psychology: scientific proof of the effectiveness of psychotherapy*(translation of the Portuguese title)^2^ is a pioneering work written by several hands, which indubitably include the most renowned specialists in the field of mental health in Brazil. We had the privilege of counting on collaboration from researchers, clinicians and educators, and this diversity was the driving force towards enrichment of the work. The text places at healthcare professionals' disposal scientific information of high methodological quality that has already been evaluated through systematic reviews published by the Cochrane Collaboration. It also provides a succinct panorama of the current evidence relating to the effectiveness of psychotherapy for treating psychiatric disorders. Each chapter presents a systematic review on the subjects (published in the Cochrane Library), with comments by one or more specialists in the field. The work also discusses issues relating to prevention of these disorders, and covers systematic reviews of studies on preventive programs at different levels. In this respect, it includes chapters on studies conducted among children and adolescents that had the aim of identifying risk factors and ways of avoiding them and protection factors and ways of promoting them.

Studies and research within the field of mental health involving children and adolescents are well-known to be very limited in numbers, despite recognition of their importance among professionals working in this field. This book also seeks to encourage evolution and expansion of this knowledge and makes suggestions for new studies.

This work can be used by healthcare professionals as a reference source in making decisions about patient care and in drawing up research protocols, thereby opening up a range of topics for scientific research evaluated by researchers in this field and by society. This is a novel text that makes a valuable contribution towards presenting research results within psychotherapy and assessing the evidence available. Its objective and instructional format facilitate access to information.
